# A cross-border outbreak of *Salmonella* Bareilly cases confirmed by whole genome sequencing, Czech Republic and Slovakia, 2017 to 2018

**DOI:** 10.2807/1560-7917.ES.2021.26.14.2000131

**Published:** 2021-04-08

**Authors:** Klára Labská, Michaela Špačková, Ondřej Daniel, Josef Včelák, Veronika Vlasáková, Tomáš Černý, Tereza Gelbíčová, Martina Florianová, Renáta Karpíšková, Dagmar Gavačová, Mária Štefkovičová, Hana Orlíková

**Affiliations:** 1 National Institute of Public Health, Prague, Czech Republic; 2 ECDC Fellowship Programme, public health microbiology path (EUPHEM), European Centre for Disease Prevention and Control (ECDC), Stockholm, Sweden; 3 Department of Molecular Endocrinology, Institute of Endocrinology, Prague, Czech Republic; 4 Food Safety Division, State Veterinary Administration, Prague, Czech Republic; 5 National Reference Laboratory for salmonella, State Veterinary Institute Prague, Prague, Czech Republic; 6 Veterinary Research Institute, Brno, Czech Republic; 7 National Reference Centre for Salmonellosis, Public Health Authority of the Slovak Republic, Bratislava, Slovakia; 8 Public Health Authority of the Slovak Republic, Regional Public Health Authority, Trenčín, Slovakia

**Keywords:** *Salmonella* Bareilly, outbreak, WGS, powdered egg product, spray dryer, food- and water-borne diseases

## Abstract

In August 2017, an increased incidence of *Salmonella* Bareilly was detected in the Czech Republic. An investigation was conducted with Slovakia to confirm the outbreak and identify the source. Probable outbreak cases were defined as cases with laboratory-confirmed *S.* Bareilly reported in either of the national surveillance systems, and/or the Czech and Slovak National Reference Laboratory databases from July 2017. Confirmed cases had the pulsed-field gel electrophoresis (PFGE) outbreak pulsotype or up to 5 alleles difference from outbreak cluster members by core genome multilocus sequence typing (cgMLST). PFGE and whole genome sequencing were used for isolate comparison. The same trawling questionnaire was used in both countries. By the end of October 2018, 325 cases were identified. Among 88 human *S.* Bareilly isolates analysed by PFGE, 82 (93%) shared an identical pulsotype; cgMLST of 17 *S.* Bareilly human isolates showed 1–2 allele difference. The trawling questionnaire excluded consumption of unusual or imported foods. In September 2018, an isolate closely related to the outbreak isolates was identified in a powdered egg product. A spray dryer was recognised as the contamination source and the production plant was closed. Using molecular typing methods, we detected a diffuse cross-border outbreak caused by *S.* Bareilly.

## Background

*Salmonella* spp. are the third most common cause of bacterial food-borne illnesses worldwide [1] and the second most commonly reported zoonotic agents in the European Union (EU) [2]. The bacterial genus *Salmonella* consists of *Salmonella enterica* and *Salmonella bongori* species. More than 2,500 serotypes of *Salmonella enterica* have been identified so far [3], many of them commonly infecting animals (e.g. poultry, pigs, cattle) and humans. The distribution of predominant serovars in each country are affected by changes in the global food and livestock trade, international travel, and human migration [4].

*Salmonella enterica* subsp. *enterica* serovar Bareilly (*S.* Bareilly) belongs to the C1 serogroup (antigenic formula 6, 7, 14: y: 1,5) and was first identified in India in 1928 [5]. In the United Kingdom (UK), 31% of all *S.* Bareilly human cases identified between 2005 and 2009 were attributed to travel from India [6]. Since 2004, *S.* Bareilly has most commonly been isolated from spices [7]. Contaminated mung bean seeds were the likely source of a *S.* Bareilly outbreak in the UK in 2010, with total of 231 cases [6]. In an outbreak of salmonellosis in the United States, which comprised 410 cases of *S.* Bareilly across 28 states and the District of Columbia, tuna scrape imported from India was identified to be the source using whole genome sequencing (WGS)-based methods [7].

Since 2016, *S.* Bareilly has been among the top 20 *Salmonella* serotypes associated with human diseases in the European Union/European Economic Area (EU/EEA) [2]. Between 2006 and 2016, *S.* Bareilly was among the top 25 serotypes detected in humans in the Czech Republic [8], with the annual incidence ranging from 0.04 to 0.23 per 100,000 inhabitants (data from the Czech national electronic communicable diseases notification system). According to data from the Czech national control programme for *Salmonella* in poultry, *S.* Bareilly was identified in broiler flocks with a prevalence of up to 0.06% [9,10].

Salmonellosis has been a mandatory notifiable disease in both the Czech Republic and Slovakia since 1951. Regional public health officers notify case-based data to the national electronic communicable diseases notification system (EpiDat/ISIN in the Czech Republic and the Epidemic Intelligence Information System (EPIS) in Slovakia). Both systems record data on all cases that meet the definition of a confirmed case in accordance with the European Commission Implementing Decision 2119/98/EC [11]. The information on *Salmonella* serovar, which is provided by routine microbiological laboratories handling human samples, is included in the reporting systems. These laboratories typically test for a limited spectrum of serovars only, and *S.* Bareilly is usually not included. The Czech and Slovak National Reference Laboratories (NRLs) (the Czech NRL is a part of the National Institute of Public Health in Prague, the Slovak NRL is part of the Public Health Authority in Bratislava) provide serotyping of less common serovars and confirm results from routine microbiological laboratories on request.

There are several options to confirm the relatedness of *Salmonella* isolates in laboratories. Macro-restriction analysis followed by pulsed-field gel electrophoresis (PFGE) is usually a suitable method for the detection and investigation of *Salmonella* outbreaks. However, in some cases, it does not provide sufficient discriminatory power to distinguish outbreak isolates [12]. Therefore, WGS-based typing methods are now increasingly applied as molecular epidemiology tools to assist in outbreak investigations [13].

## Outbreak detection

In August 2017, the number of human *S.* Bareilly cases reported in the Czech Republic exceeded the maximal annual total number of cases from the previous 5 years (n = 25). This was accompanied by an increased number of requests from routine microbiological laboratories to the NRL for *S.* Bareilly serotype identification/confirmation.

The outbreak was recognised in the Czech Republic in October 2017 after identifying the outbreak PFGE pulsotype (as described in the Results section) in selected *S.* Bareilly isolates from the Czech NRL isolate collection. This testing was outsourced to the Veterinary Research Institute (VRI), Brno, Czech Republic, which provides PFGE testing for the State Veterinary Administration (SVA), Czech Republic, and the Czech and Slovak public health sectors.

Following a personal communication between the Czech and Slovak NRLs and the VRI, the Slovak NRL carried out testing, through the VRI, of all Slovak *S.* Bareilly isolates collected during 2017. In November 2017, the VRI detected the outbreak PFGE pulsotype in *S.* Bareilly isolates from Czech and Slovak cases collected by both NRLs. The outbreak cluster was later confirmed by the core genome multilocus sequence typing (cgMLST) analysis (for details of PFGE and cgMLST data and their interpretation see Methods and Results).

In November 2017, the Czech NRL passed the information about the ongoing outbreak to the SVA in order to start the investigation of a probable food/feed *S.* Bareilly source. As a result, all available *S.* Bareilly isolates from the Czech national control programme for *Salmonella* in poultry for the period of 2016–17 were screened by PFGE. Later, as the outbreak continued, the concerned parties also communicated the findings through the Epidemic Intelligence Information System for Food- and Waterborne Diseases and Zoonoses (EPIS-FWD) platform administered by the European Centre for Disease Prevention and Control (ECDC).

We conducted a national and a subsequent international outbreak investigation with Slovakia to confirm and control the outbreak and to identify its source.

## Methods

### Outbreak case definition

A probable outbreak case was defined as laboratory-confirmed salmonellosis (fulfilling the Decision 2119/98/EC criteria [11]) caused by *S.* Bareilly with symptom onset from 1 July 2017, in either the Czech Republic or Slovakia.

A confirmed case was defined as a probable case with the PFGE outbreak pulsotype or a probable case with up to 5 alleles of difference from the outbreak cluster members by cgMLST [14], in either the Czech Republic or Slovakia.

### Case finding and case management

The case-based data from the Czech NRL and EpiDat/ISIN were merged into a joint database that was used for the outbreak line list and further descriptive analysis of the Czech outbreak cases. The line list of Slovak cases was created identically but handled separately. Laboratory databases and mandatory notification surveillance systems were the only sources of case identification. Contact tracing was performed by regional public health officers as a part of routine investigation of the cases.

Standard precautions against further spreading of salmonellosis were implemented by the regional public health authorities. Food handlers, sources of drinking water and waste water management in households were targeted.

In January 2018, after the outbreak had been confirmed, an outbreak notification was sent to all 14 regional public health epidemiology offices in the Czech Republic. An informative letter was also sent to the microbiological laboratories taking part in the external quality assessment organised by the National Institute of Public Health, with a request to send all C1 serogroup isolates to the Czech NRL. New *S.* Bareilly cases confirmed by the Czech NRL were immediately reported to regional epidemiologists via a phone call requesting them to apply the trawling questionnaire (see below).

Information about the outbreak was shared with the food and veterinary authorities at regular meetings.

Information about the outbreak was also shared on the ECDC’s EPIS-FWD communication platform; at first, as a response to urgent inquiry number 427 (a cluster of *S.* Bareilly in Finland) and later as urgent inquiry number 472, which was created by the Czech Republic on 21 April 2018. Slovakia then responded to urgent inquiry number 472.

### Microbiological investigation and whole genome sequencing

Serotyping is the only typing method used in routine microbiological laboratories in both countries, and the panel of the antisera used is limited. Routine laboratories send isolates for further characterisation to the NRLs on a voluntary basis.

We selected 59 Czech and 32 Slovak human *S.* Bareilly isolates for PFGE analysis. These isolates were selected from all *S.* Bareilly isolates collected by both NRLs between 1 January and 31 October 2017 and covered all Czech and Slovak regions and age groups. The PFGE analysis was performed repeatedly in both a prospective and retrospective manner. WGS data were available for 17 Czech isolates from the period of 1 July 2017 to 10 December 2017. This analysis was supported by the ECDC FWD exchange programme.

Poultry isolates were obtained via the Czech national control programme for *Salmonella* in poultry conducted by SVA (2016–17, n = 5), and 2 additional isolates were obtained from the Czech VRI repository (2016–17, obtained through research activities).

PFGE analysis was performed by both NRLs (human isolates and a food isolate) and the Czech VRI (Czech and Slovak human, poultry and environmental isolates) following the PulseNet protocol [15] using the *Xba*I enzyme. Similarity analysis was performed using Dice coefficient with 1.0% band position tolerance. We kept strict rules for the outbreak pulsotype with 0 band difference and 1.10% band optimisation, and the isolates were separated into similarity clusters by the unweighted-pair group method using average linkages. One band difference in the PFGE pattern was considered a different pulsotype.

DNA for WGS was extracted using the QiaAmp DNA mini kit (Qiagen, Hilden, Germany) according to the manufacturer's instructions and sent to the ECDC outsourced laboratory for WGS. Data analysis was performed with the EnteroBase Backend Pipeline for cgMLST [16] and Enterobase single nucleotide (nt) polymorphism (SNP) typing pipeline for the construction of the RaxML tree [17] using FigTree v. 1.4.4 (http://tree.bio.ed.ac.uk/software/figtree/) for visualisation [18]. The RaxML tree was computed based on 3,067 variant sites found in non-repetitive regions that are present in 90% or more of all the queried genomes.

### Routine epidemiological screening reports

All *S.* Bareilly cases reported in the Czech Republic in 2017 were interviewed on a routine basis by a public health epidemiologist. The interviews provide the basis for filing notifications via the electronic reporting system, but a separate paper record of each interview is also maintained. The paper record is an open text document that usually includes additional data such as exposure and symptoms. We analysed 97 case-based *S.* Bareilly epidemiological records from 1 July to 31 December 2017 from 11 of the 14 regions of the Czech Republic (including all available records from regional public health offices that accounted for 55% of the reported cases). In order to search for a possible common exposure and to better understand the symptoms of the disease caused by *S.* Bareilly, data on food items were grouped (e.g. pork, beef, chicken, dairy products) and presented as proportion of exposed in percent.

### Trawling questionnaire

For hypothesis generation, the Salmonella_MASTER_spørgeskema_nov_2017 trawling questionnaire (Statens Serum Institut, Copenhagen, Denmark) was translated into Czech language and adapted to the local conditions, was shared with Slovakia and used in both countries. This questionnaire consists of 3 parts. The first one is dedicated to disease symptoms, household exposure, traveling, special diet and shopping habits description, the second part is meal by meal menu from the last three days before onset of symptoms and the last part is targeted on in-depth examination of food items consumed in the week before onset of symptoms. The mentioned exposures were assessed in a semi-subjective manner guided by the percentage of cases exposed. 

### Environmental and trace-back investigation

No targeted investigation in the food chain or poultry flocks was conducted during the outbreak period due to the broad hypothesis from the trawling questionnaires, which did not allow identification of any particular food item as a possible outbreak source/vehicle.

The SVA, which is responsible for the quality control of all products of animal origin produced in the Czech Republic, identified a *S.* Bareilly isolate closely related to the human outbreak isolates, in a powdered egg product (dried egg melange, see below). As this indicated a potential relationship between the egg product and the outbreak, the SVA authority repeatedly visited and investigated the company producing the powdered egg. The investigation included administrative inspections in farms that produced eggs for further processing (with the focus on the adherence to the practices directed by the national control programme for *Salmonella* in poultry), and the microbiological screening of company employees.

### Ethical statement

Ethical approval to conduct the study was not needed. In the Czech Republic and Slovakia, investigation of foodborne outbreaks is a legal obligation.

## Results

**Figure 1:**
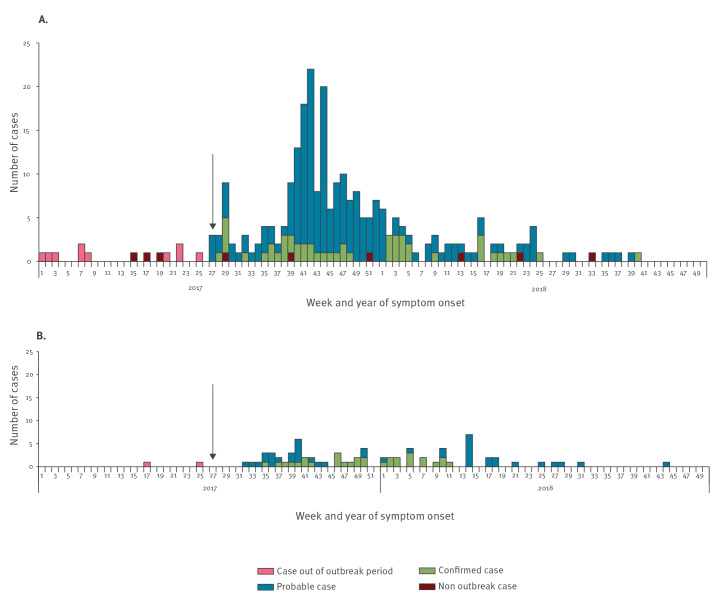
Distribution of probable and confirmed cases of *Salmonella* Bareilly according to case definition, by week of symptom onset in (A) Czech Republic and (B) Slovakia, 1 January 2017–31 December 2018 (n = 325)

The first confirmed *S.* Bareilly case detected with the outbreak pulsotype had disease onset in July 2017. Therefore, we assumed the outbreak started in July 2017 (Figure 1A). The number of cases peaked in October 2017, and the last confirmed case was reported in October 2018. From 1 July 2017 to 31 October 2018, 250 probable outbreak cases were identified in the Czech Republic (Figure 1A). Of these 250 probable cases, 56 (22%) were tested by molecular typing methods, and six (2%) were excluded as non-outbreak cases because of different pulsotypes. A total of 13 *S.* Bareilly cases were detected outside the outbreak period, of which three isolates differed from the outbreak pulsotype (Figure 1A).

In Slovakia, 75 probable cases were identified during the outbreak period, and 32 (43%) of these were tested by PFGE. The results showed that all 32 belonged to the outbreak type. Two small peaks were observed: the first in October 2017 and the second in April 2018. The last probable case was reported in October 2018 (Figure 1B).

**Figure 2:**
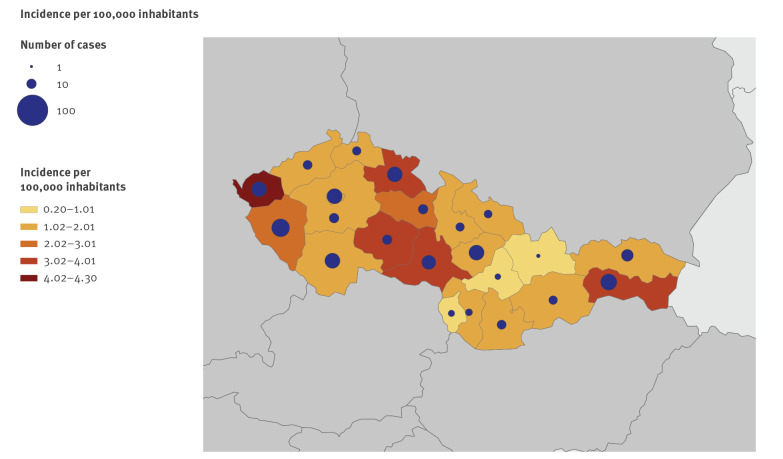
Map displaying the numbers of *Salmonella* Bareilly cases and the notification rates by region, Czech Republic and Slovakia, 1 July 2017–31 October 2018 (n = 325)

The incidence from 1 July 2017 to 31 October 2018 for all Czech regions (n = 14) and all Slovak regions (n = 8) is shown in Figure 2. The overall incidence was 2.4 cases per 100,000 inhabitants (range by region: 5–28) for the Czech Republic, and 1.4 cases per 100,000 inhabitants (range by region: 7–35) for Slovakia. The weekly number of cases in each region did not exceed five.

**Figure 3:**
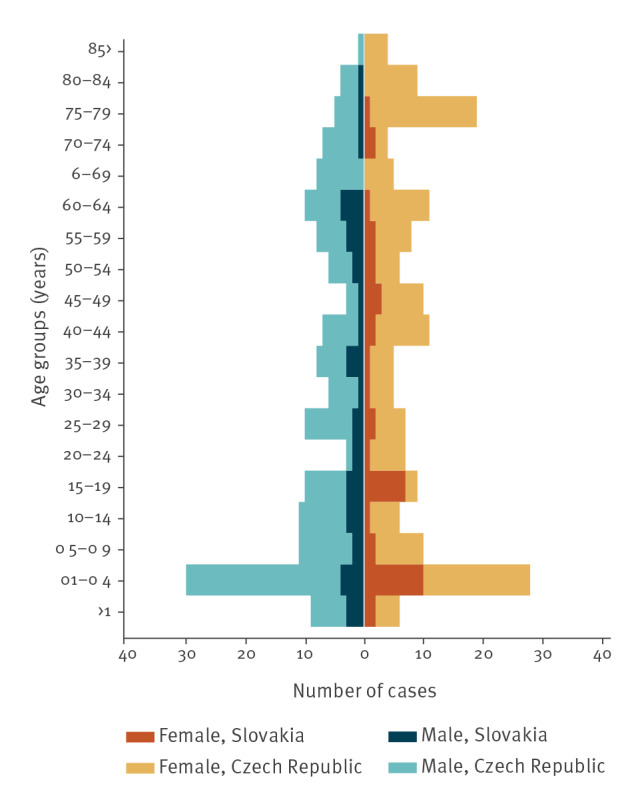
Age and sex distribution of *Salmonella* Bareilly probable and confirmed outbreak cases, Czech Republic and Slovakia, 1 July 2017–31 October 2018 (n =325)

All age groups were affected in both countries (Figure 3). The highest number of cases was reported in the 1–4 years age group. Similar proportions of males and females were observed among the cases (Figure 3). Epidemiological investigations did not detect any epidemiological link between the cases. Only six small family clusters of 2–4 household members and two mother and newborn case pairs were identified. Travelling abroad was only reported in a single Czech case.

### Outcomes and severity of symptoms

No death due to the outbreak was reported in either country.

Of 224 Czech cases where data on hospitalisation were available, 69 (31%) required hospitalisation. Hospitalised cases were present in all age groups. Data on symptoms were available from routine epidemiological screening reports for 82 cases. The most prevalent symptoms were diarrhoea (93%), fever (50%), vomiting (21%), and abdominal cramps (28%). In Slovakia, 23 (31%) of 75 cases were admitted to hospital and no data on symptoms were available.

### Trawling questionnaire

From January 2018 to April 2018, 14 Czech and four Slovak confirmed cases were interviewed. All respondents reported they had consumed chicken, eggs, ham, and yogurt. The respondents bought their food predominantly in grocery chains; seven different grocery chains were mentioned. The respondents did not report consumption of freshwater fish or seafood products, unusual or imported food items, seasoning with fresh herbs, sprouts, or seeds. Median time from symptom onset to interview was 19.5 days and ranged between 10 and 40 days.

After the outbreak pulsotype had been recognised in a *S.* Bareilly isolate originating from a powdered egg product (see below), the data were re-analysed with respect to the ingredients present in finished products consumed by the respondents. Previous consumption of some products containing powdered eggs was reported by all respondents (data not shown).

### Molecular typing-pulsed-field gel electrophoresis and whole genome sequencing

**Figure 4:**
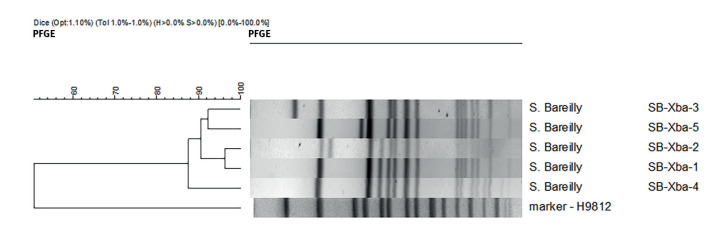
The dendrogram of detected *Salmonella* Bareilly PFGE pulsotypes, Czech Republic and Slovakia, 2016–2018

The results of pulsed-field gel electrophoresis (PFGE) revealed five different pulsotypes, SB-Xba 1–5, during 2016–18, with SB-Xba 1 being identified as the outbreak pulsotype (Figure 4). Isolates from 50 human cases in the Czech Republic, 32 human cases in Slovakia and the powdered egg product isolate shared the identical PFGE outbreak pulsotype SB-Xba-1. According to the EPIS-FWD UI 427 and 472 communications, this pulsotype was not reported in any other EU/EEA country. The other four observed pulsotypes differed from the outbreak isolate by 1–3 bands (Figure 4) and were dispersed over both countries.

SB-Xba-1 is the pulsotype associated with the outbreak.

**Figure 5:**
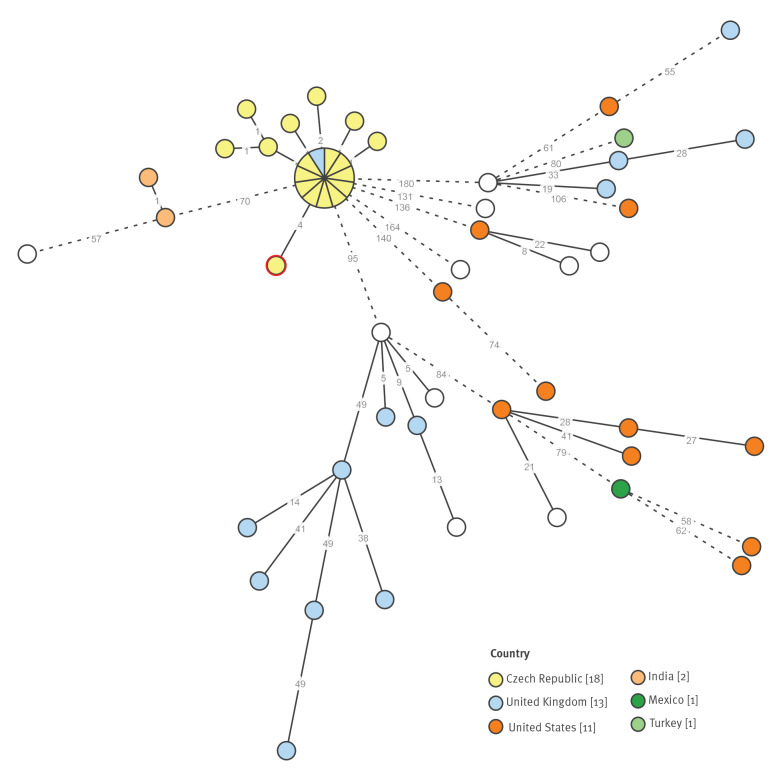
The cgMLST minimum spanning tree based on Czech outbreak isolates (indicated in yellow) and strains belonging to cgMLST type HC_200_-21660 (56 strains in total) from the EnteroBase, 22 January 2019

Whole genome sequencing was performed on 17 human *S.* Bareilly isolates from the outbreak period and the powdered egg product isolate. All isolates, including those from the powdered egg product, formed a cluster (Figure 5, highlighted in yellow) that had a between-isolates distance of maximum six alleles. The distance of this cluster from the other isolates was at least 70 alleles. One congruent isolate belonging to this cluster was found in the UK during the outbreak period. Any epidemiological link of this single case to the outbreak is unknown because of the lack of metadata on exposure and travel history in the EnteroBase database.

The Czech powdered egg product isolate is indicated by yellow circle with a red border.

Strains from different countries are distinguished by different colours of circles.

Colourless circles indicate strains with missing information on the country of origin.

Eleven isolates from the cgMLST cluster were also analysed by PFGE. All of them were found to belong to the SB-Xba-1 pulsotype.

**Figure 6:**
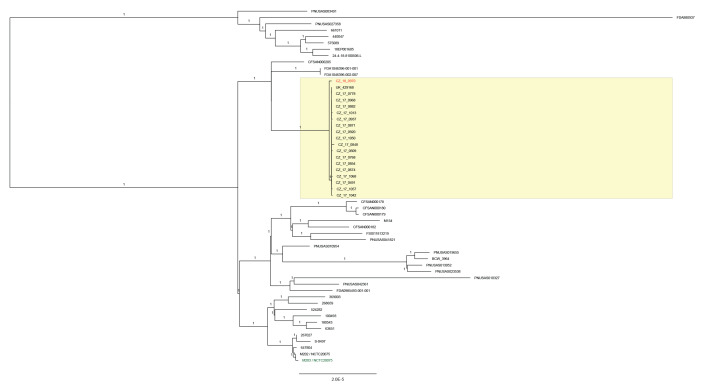
SNP- based phylogeny of *Salmonella* Bareilly strains (the same dataset as used for cgMLST analysis) using the SNP-Project in EnteroBase, 21 January 2019

The phylogenetic analysis of *S.* Bareilly isolates from the outbreak cases and *S.* Bareilly strains available in the EnteroBase (a maximum difference of 200 alleles, HC _200_-21660) demonstrated that the outbreak isolates formed a cluster within a five SNPs distance. The powdered egg product isolate (CZ_18_970) diverged earlier but still fell within a six SNPs cluster (Figure 6). The phylogenetic analysis thus supported a close relationship between the isolate from the powdered egg product and the human outbreak isolates, which all formed a monophyletic cluster (Figure 6).

Isolates from the outbreak, including human cases and the powdered egg product, are depicted in the yellow box.

The powdered egg product isolate is indicated in red letters.

The reference genome of *Salmonella* Bareilly (M203 / NCTC20075) is indicated in green letters.

The scale bar represents the number of substitutions per site.

## Outbreak source location and control measures

A spray dryer was identified as the source of contamination. In July 2018, a spray dryer was moved from its previous location (location A under company A) to a new location (location B under company B) and production of testing batches of dried eggs was started at location B. The testing batches had an unsatisfactory sensory quality, therefore company B ordered testing of their products. During this testing, *S.* Bareilly was detected in a powdered egg product produced in July 2018; the contamination of the product was recognised in September 2018. These batches of product were not marketed.

The sprayer had been operating at location A, where the last batch of dried egg products (250,000 kg) was produced in March 2017. The batch had an expiration period of 18 months. It was stored at location A and was sold until July 2018 to food producers across both countries and used as a food ingredient until the expiry date, without the opportunity to trace back. Company A went out of business in 2017 and the spray dryer used in the production of the dried egg products remained there until July 2018 when it was moved to location B.

After recognising the contaminated product at location B, two inspection visits of company B were performed by SVA. Environmental samples from the premises (the spray dryer, working area surfaces, utensil for preparation of ready-to-eat catering products and catering products - mayonnaise, ham hock, baked flank steak, hamburger, schnitzel) and from employees were taken.

The first visit took place in September 2018. Neither *S.* Bareilly nor a violation against the hazard analysis and critical control points (HACCP) guidelines were detected, but active screening of powdered egg product batches was started. The spray dryer underwent a repeated complete cleaning and disinfection. Batch controls were regularly performed from September 2018 until July 2019 and *S.* Bareilly-positive powdered egg products were repeatedly identified. At the end of June 2019, a second inspection visit, including an extensive environmental testing, confirmed a massive contamination of the whole technology of the spray dryer at location B. In July 2019, all powdered egg products produced since January 2019 were recalled from the market and the facility was closed.

## Discussion

The Czech Republic and Slovakia report the highest numbers of salmonellosis cases in the EU/EEA, but there is a lack of evidence of the sources/vehicles of food-borne outbreaks compared with other EU/EEA countries [2]. Both the Czech Republic and Slovakia have a two-level system of public health control (a regional and a national) whereby the national level serves as a risk assessor and has to be invited by the supervising regional authority to support an outbreak investigation. The outbreak reported here was dispersed over all 14 Czech regions and all eight Slovak regions, with low numbers of cases per region. Therefore, the outbreak was recognisable only at national level, resulting in late outbreak recognition. A simple descriptive epidemiological analysis of the line list including age and sex was insufficient in a hypothesis construction. We revealed gaps in the currently used interviewing strategy that has so far been focused on egg and chicken meat consumption to identify food-borne outbreak sources. Therefore, we had to find and adopt the most efficient strategy for case finding and confirmation under local conditions. Specifically, we applied the trawling questionnaire in both affected countries for the first time. However, the limitation of this approach was the late application of the questionnaire during the outbreak period, i.e. after the outbreak peak when the number of new cases was already low. As a result, the number of trawling questionnaire respondents was low, although the response rate was high (75%). As the probable cause of the outbreak was present as a common ingredient in multiple food types, the trawling questionnaire did not provide a clear indication regarding the contaminated food item. However, the questionnaire helped us understand that the vehicle was probably widely distributed by grocery chains throughout both countries and was not linked to any unusual or imported food items. We cannot rule out a recall bias due to the time delay from symptom onset to the interview.

We decided not to conduct an analytical study because of the low number of new cases and the weak hypothesis based on the trawling questionnaire. An increased awareness among food and veterinary authorities later led to the identification of the closely related *S.* Bareilly isolate in the powdered egg product (dried egg melange).

After the recognition of the genetically closely related isolate in the powdered egg product, the trawling questionnaire data were re-evaluated and a possible exposure to food items containing powdered egg products was found in all cases. The range of the suspected food items was very broad (from chocolate bars to mayonnaise), and their brands remained unidentified in more than half of cases (data not shown).

The use of WGS-based typing methods led to many successful trace-backs in multi-country outbreaks [19,20]. Such outbreaks are challenging to detect as it is hard to confirm whether there is an epidemiological link between cases [21]. Although routine serotyping of *Salmonella* isolates can be beneficial in the initial phase of outbreak detection, especially when the serotype is rare, more precise typing methods are required for outbreak confirmation. The only advanced typing method available in both countries was PFGE. As the resources allocated for such events were limited, we were not able to test all human isolates. All our sequenced human isolates originated from the Czech Republic in 2017.

PFGE of *S.* Bareilly has been reported to have a low level of discrimination, especially for strains originating in South Asia [13]. However, in the outbreak reported here, a specific pulsotype for Czech and Slovak outbreak isolates was identified. Different pulsotypes were observed prior to, and also during, the outbreak period (Figures 1 and 4). According to the EPIS-FWD UI 472 ECDC Summary, the outbreak pulsotype was not reported by any other EU/EEA country.

PFGE data were congruent with the results of cgMLST. In addition, all tested Czech human and powdered egg product isolates formed a separate monophyletic cluster using the SNP analysis of variant sites (RAxML phylogeny). The observed distance in SNP phylogeny between the human outbreak isolates and the powdered egg isolate may be due to the one-year gap between sampling the human cases and the powdered egg products.

Powdered egg products are considered safe due to their microbicidal treatment (thermal treatment, low water activity), but contaminated spray dryers have occasionally been described as outbreak sources [22]. Powdered egg products (egg yolk, egg white, or a mixture of both) serve as ingredients of multiple food products and additional heat inactivation is not required.

The owner of the spray dryer during the outbreak period at location A had a large distribution network across both countries including bakeries, semi-finished product producers and confectionery producers, which is in accordance with the countrywide distribution of the cases. All the batches were sold out and consumed at the time the closely related isolate in the powdered egg products was detected. The current owner of the spray dryer at location B cooperated with the state veterinary authorities to solve the problem of contamination. The facility has been closed and a complete rebuilding of the technology is ongoing.

### Conclusion

Using molecular typing methods enabled us to link *S.* Bareilly cases distributed across two countries over a prolonged time period. PFGE served successfully as the first line tool in the outbreak identification, and WGS-based methods (cgMLST and SNP analyses) played a crucial role in the confirmation of the outbreak and in the identification of the possible source. The adopted trawling questionnaire was shared by both affected countries and can be used in future investigations of suspected food-borne outbreaks in a timely manner. Contaminated spray dryers should be considered potential sources for cross-contamination of powdered egg products with *Salmonella*.

## References

[1] Pires SM Fischer-Walker CL Lanata CF Devleesschauwer B Hall AJ Kirk MD Aetiology-specific estimates of the global and regional incidence and mortality of diarrhoeal diseases commonly transmitted through food. PLoS One. 2015;10(12):e0142927. 10.1371/journal.pone.0142927 26632843 PMC4668836

[2] European Food Safety Authority and European Centre for Disease Prevention and Control (EFSA and ECDC). The European Union summary report on trends and sources of zoonoses, zoonotic agents and food-borne outbreaks in 2017. EFSA J. 2018;16(12):e05500. 32625785 10.2903/j.efsa.2018.5500 PMC7009540

[3] Grimont PAD, Weill F-X. Antigenic formulae of the Salmonella servovars: WHO collaborating centre for reference and research on Salmonella. 9th Edition, Institute Pasteur. 2007.

[4] Hendriksen RS Vieira AR Karlsmose S Lo Fo Wong DMA Jensen AB Wegener HC Global monitoring of Salmonella serovar distribution from the World Health Organization Global Foodborne Infections Network Country Data Bank: results of quality assured laboratories from 2001 to 2007. Foodborne Pathog Dis. 2011;8(8):887-900. 10.1089/fpd.2010.0787 21492021

[5] Bridges RF Scott WM. A new organism causing paratyphoid fever in India. J R Army Med Corps. 1931;56:241-9.

[6] Cleary P Browning L Coia J Cowden J Fox A Kearney J A foodborne outbreak of Salmonella Bareilly in the United Kingdom, 2010. Euro Surveill. 2010;15(48):8. 10.2807/ese.15.48.19732-en 21144449

[7] Hoffmann M Luo Y Monday SR Gonzalez-Escalona N Ottesen AR Muruvanda T Tracing origins of the Salmonella Bareilly strain causing a food-borne outbreak in the United States. J Infect Dis. 2016;213(4):502-8. 10.1093/infdis/jiv297 25995194

[8] Dědičová D, Pihávková H, Mašková J. Humánní kmeny salmonel identifikované v NRL pro salmonely v průběhu roku 2008. [Human strains of Salmonella identified in the NRL for Salmonella in 2008]. Zprávy CEM (SZÚ, Praha) 2009; 18(5):164-7. Czech. Available from: http://www.szu.cz/uploads/documents/CeM/Zpravy_EM/18_2009/5_kveten/164salm.pdf

[9] Státní veterinární správa (State Veterinary Administration). Národní programy tlumení salmonel – Metodika kontroly zdraví a nařízené vakcinace na rok 2019. [National programmes for Salmonella control - methods for health control and ordered vaccination in 2019]. Prague: The State Authority; 2019. [Accessed: 22 Feb 2019]. Czech. Available from: https://www.svscr.cz/zdravi-zvirat/programy-tlumeni-vyskytu-salmonel/

[10] European Commission. Commission implementing decision (EC) No 517/2011 of 25 May 2011 implementing Regulation (EC) No 2160/2003 of the European Parliament and of the Council as regards a Union target for the reduction of the prevalence of certain Salmonella serotypes in laying hens of Gallus gallus and amending Regulation (EC) No 2160/2003 and Commission Regulation (EU) No 200/2010. Official Journal of the European Union. Luxembourg: Publications Office of the European Union. 26 May 2011. Available from: https://eur-lex.europa.eu/eli/reg/2011/517/oj

[11] European Commission. Commission implementing decision (EU) No 506/2012EU of 8 August 2012 amending Decision 2002/253/EC laying down case definitions for reporting communicable diseases to the Community network under Decision No 2119/98/EC of the European Parliament and of the Council (notified under document C (2012) 5538). Official Journal of the European Union. Luxembourg: Publications Office of the European Union. 27 Sep 2012. Available from: https://eur-lex.europa.eu/LexUriServ/LexUriServ.do?uri=OJ:L:2012:262:0001:0057:EN:PDF

[12] Chiou CS Lin JM Chiu CH Chu CH Chen SW Chang YF Clonal dissemination of the multi-drug resistant Salmonella enterica serovar Braenderup, but not the serovar Bareilly, of prevalent serogroup C1 Salmonella from Taiwan. BMC Microbiol. 2009;9(1):264. 10.1186/1471-2180-9-264 20017951 PMC2806260

[13] Nadon C Van Walle I Gerner-Smidt P Campos J Chinen I Concepcion-Acevedo J PulseNet International: Vision for the implementation of whole genome sequencing (WGS) for global food-borne disease surveillance. Euro Surveill. 2017;22(23):30544. 10.2807/1560-7917.ES.2017.22.23.30544 28662764 PMC5479977

[14] Besser JM Carleton HA Trees E Stroika SG Hise K Wise M Interpretation of whole-genome sequencing for enteric disease surveillance and outbreak investigation. Foodborne Pathog Dis. 2019;16(7):504-12. 10.1089/fpd.2019.2650 31246502 PMC6653782

[15] Centre for Disease Control and Prevention (CDC). Standard operating procedure for PulseNet PFGE of Escherichia coli O157:H7, Escherichia coli non-O157 (STEC), Salmonella serotypes, Shigella sonnei and Shigella flexneri; Atlanta: CDC; 2013. PNL05 Last Updated December 2017. Available from: http://www.cdc.gov/pulsenet/pdf/ecoli-shigella-salmonella-pfge-protocol-508c.pdf

[16] Zhou Z, Alikhan NF, Sergeant MJ, Luhmann N, Vaz C, Francisco AP, et al. Grapetree: Visualization of core genomic relationships among 100,000 bacterial pathogens. Genome Res. 2018; bioRxiv. 10.1101/gr.232397.117 PMC6120633 30049790

[17] Stamatakis A. RAxML version 8: a tool for phylogenetic analysis and post-analysis of large phylogenies. Bioinformatics. 2014;30(9):1312-3. 10.1093/bioinformatics/btu033 24451623 PMC3998144

[18] Rambaut A. FigTree version 1.3.1. 2009. Available from: http://tree.bio.ed.ac.uk.

[19] Meinen A Simon S Banerji S Szabo I Malorny B Borowiak M Salmonellosis outbreak with novel Salmonella enterica subspecies enterica serotype (11:z41:e,n,z15) attributable to sesame products in five European countries, 2016 to 2017. Euro Surveill. 2019;24(36):1800543. 10.2807/1560-7917.ES.2019.24.36.1800543 31507266 PMC6737830

[20] Pijnacker R Dallman TJ Tijsma ASL Hawkins G Larkin L Kotila SM An international outbreak of Salmonella enterica serotype Enteritidis linked to eggs from Poland: a microbiological and epidemiological study. Lancet Infect Dis. 2019;19(7):778-86. 10.1016/S1473-3099(19)30047-7 31133519

[21] Dallman T Inns T Jombart T Ashton P Loman N Chatt C Phylogenetic structure of European Salmonella Enteritidis outbreak correlates with national and international egg distribution network. Microb Genom. 2016;2(8):e000070. 10.1099/mgen.0.000070 28348865 PMC5320589

[22] Cahill SM Wachsmuth IK Costarrica ML Ben Embarek PK. Powdered infant formula as a source of Salmonella infection in infants. Clin Infect Dis. 2008;46(2):268-73. 10.1086/524737 18171262

